# Radionuclide Treatment with 153Sm-EDTMP is Effective for the Palliation of Bone Pain in the Context of Extensive Bone Marrow Metastases: A Case Report

**Published:** 2014

**Authors:** Kalevi Kairemo, Nigora Rasulova, Justina Suslaviciute, Tuomo Alanko

**Affiliations:** 1 Department of Molecular Radiotherapy & Nuclear Medicine, Docrates Cancer Center, Helsinki, Finland; 2 Department of Medical Oncology, Docrates Cancer Center, Helsinki, Finland

**Keywords:** ^153^Sm-EDTMP, Bone marrow metastases, Bone metastases, Positron emission tomography (PET), Radionuclide therapy, Small cell lung cancer

## Abstract

Radionuclide therapy is widely used as an effective modality in the management of bone pain. The main indication for this treatment is symptomatic bone metastases, confirmed by bone scintigraphy. We present a case of small cell lung cancer (SCLC) stage T_4_N_2_M_1b_, with a good metabolic response to systemic therapy and radiotherapy of the primary tumor and locoregional disease, which became metabolically less active and remarkably smaller in size (reduction to 1/6 of the original volume). In spite of the good overall response, the patient developed a syndrome with severe bone pain and had progression in the bone marrow metastases, confirmed by ^18^F-FDG PET/CT. The patient received ^153^Sm-EDTMP treatment with a good clinical response. However, in the whole body bone scan with the therapeutic dose, there was no visual evidence of bone metastasis. Retrospectively, by drawing the region of interest, it was possible to identify one metastatic site. The possible mechanisms of the efficacy of this treatment modality, in this specific setting, are also discussed.

## Introduction

Small cell lung cancer (SCLC) is an aggressive form of lung cancer, with a poor prognosis and post-mortem evidence of bone metastasis in up to 36% of patients. Bone marrow micrometastases are found in 22-60% of affected individuals (1). Up to 60-90% of patients with bone metastases experience severe bone pain in the terminal stages of their disease.

Radionuclide therapy is widely used as an effective modality in the management of bone pain. Bone-seeking radiopharmaceuticals such as samarium-153-ethylene diamine tetramethy-lene phosphonate (^153^Sm-EDTMP), strontium-89 chloride (^89^SrCl_2_), and radium-223 chloride (^223^RaCl_2_) have affinity to skeletal tissue and accumulate in areas of increased bone turnover after intravenous administration.

The indication for radionuclide therapy is presence of osteoblastic or mixed metastases, confirmed by bone scan (2). However, bone marrow metastatic disease can be completely negative in bone scan. ^18^F-FDG PET/CT in patients with SCLC has been shown to have prognostic significance and may help in decision-making for the therapeutic management of limited-stage disease (3, 4). Herein, we report a case in which SCLC bone marrow metastases, confirmed by ^18^F-FDG PET/CT, could be successfully treated with radionuclide therapy.

## Case report

A 67-year-old man presented with excruciating pain in the right side of the thorax and his back. In diagnostic imaging, tumor masses in the right lung, pleura and paraspinal regions were detected. Pleural tumor was biopsied, and histopathology represented SCLC. TNM-staging according to AJCC/UICC was T_4_N_2_M_1b_.

**Figure 1:**
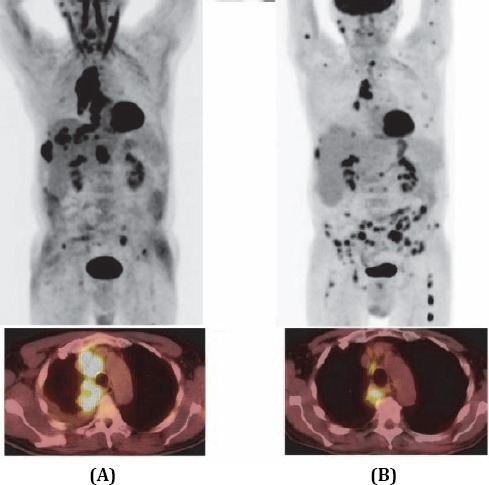
^18^F-FDG PET/CT scan before and after therapy. A. At the initial staging, B. Response to the therapy of primary tumor and locoregional disease

Figure 1A shows ^18^F-FDG PET/CT scan before therapy at initial staging. ^18^F-FDG PET/CT showed a large lobulated tumor mass adjacent to the right pulmonary hilum, and the upper mediastinum demonstrated strong fluorodeoxyglucose (FDG) uptake [maximum standardized uptake value (SUV_max_) of 10.5; maximum dimension of 10cm)]. There were multiple pathological lymph nodes (including pretracheal, subcarinal, right hilar, and retrocrural nodes) with SUV_max_ ranging from 7.9 to 11 (maximum dimension ranged from 3.5 to 5.5 cm).

A paraspinal tumor in the lower thoracic spine (SUV_max_ of 6.9, dimension of 12.5 cm) and multiple uptakes in parietal pleura with subpleural extension in lateral and basal regions (with SUV_max_ ranging from 6.9 to 8.7 and thickening up to 3×5 cm), protruding into the intercostal space of VIII/IX rib, were also seen. There were skeletal uptakes in the left iliac crest, both iliac wings, S1, and right ischium. SUV_max_ ranged from 2.9 to 4.7, and the size varied from 1 to 3.1 cm.

The patient received four cycles of standard chemotherapy consisting of cisplatin, etoposide, and zoledronic acid and underwent palliative irradiation to the thoracic area including the primary tumor (total dose of 26 Gy in 13 fractions). After the treatment, the patient experienced good palliation of the pain in the right side of the thorax, but started having increasing pain in the sacral bone, knee joints, and both legs.

The PET/CT scan after 4 cycles of chemotherapy (Figure 1B) demonstrated that the large lobulated tumor mass, adjacent to the right pulmonary hilum, and upper mediastinum remarkably decreased in size and ^18^F-FDG uptake (SUV_max_ decreased from 10.5 to 7.4; maximum dimension reduced from 10 cm to 5 cm; volume decreased from 200 cm^3^ to 30 cm^3^). Pathological lymph nodes (pretracheal, subcarinal, right hilar, and retrocrural) almost completely disappeared with SUV_max_ ranging from 2.9 to 3.4; maximum dimension was less than 1 cm.

Paraspinal tumor in the lower thoracic spine demonstrated SUV_max_ reduction from 6.9 to 3.2. The pleural tumor, protruding to the intercostal space, also decreased in size from 3 cm × 5 cm to 1 cm × 1 cm, and SUV_max_ decreased from 8.7 to 5.8. However, there were multiple (>20) new skeletal uptakes in both proximal humeri, right scapula, vertebrae C3, T1, L2, L3, sacrum, multiple small lesions in the pelvic girdle, and both proximal femoral bones, with SUV_max_ ranging from 3.5 to 8.7 and size from 1.5 to 11 cm; all were located in the bone marrow.

Due to bone pain, ^153^Sm-EDTMP treatment with a dose of 3552 MBq, together with a small dose of weekly docetaxel 20 mg/m^2^, was administered to the patient. One week after ^153^Sm-EDTMP administration, the patient reported significant reduction in bone pain. However, in the whole body bone scan, there were no focal bone lesions. There were some irregularities in the distribution of tracer uptake in the projection of the sacroiliac joints, but no obvious signs of bone metastatic disease were observed.

**Figure 2:**
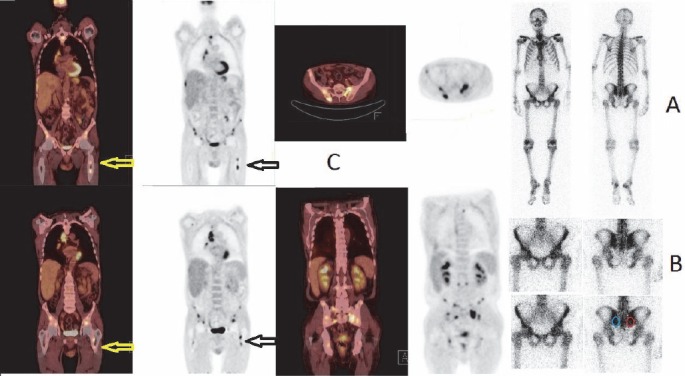
Bone marrow involvement according to ^18^F-FDG PET/CT scan and whole body bone scintigraphy 6 days after ^153^Sm-EDTMP bone pain palliation therapy

Quantitative comparison of sacroiliac joint regions of interest showed a significant difference between the left and right sacroiliac joint, with a ratio of 1.9:1.0. The regions are shown in color in the ^153^Sm-EDTMP spot images of the pelvis (Figure 2). By visual assessment, there was no difference in the pelvic region in the ^153^Sm-EDTMP images, whereas in the PET/CT fusion images, pelvic bone marrow involvement was evident (Figure 2).

## Discussion

According to the European guidelines, “^153^Sm-EDTMP is indicated in the management of bone pain due to skeletal metastases, involving more than one site associated with an osteoblastic response on bone scintigraphy” (2). Our patient did not meet these criteria, since he was affected by an extensive bone marrow disease with skeletal pain, without any significant findings in bone scintigraphy. One week after ^153^Sm-EDTMP administration, the patient experienced significant pain reduction.

It has been reported that bone mineral matrix contains numerous growth factors and cytokines, which are released during normal bone remodeling and provide a favorable microenvironment for tumor cell colonization and proliferation. Integrins, a family of cell-surface-receptors, mediate both cell-cell and cell-matrix interactions between fibroblasts, endothelial and inflammatory cells (5). Tumor cells secrete growth factors that stimulate the activity of osteoclasts and/or osteoblasts, and bone-derived growth factors reciprocally enhance the survival and proliferation of tumor cells.

The pathophysiological mechanisms of pain in patients with bone metastases are still under investigation and may include tumor-induced osteolysis, production of tumor growth factors and cytokines, nerve infiltration, ion channel stimulation, and production of endothelins and nerve growth factors in local tissues. One of the involved factors is vascular endothelial growth factor (VEGF), which may play a pivotal role in neuropathic pain. VEGF receptors (VEGFR1 and VEGFR2) are localized to vascular endothelial cells or macrophages, and their ligand VEGF-A is accumulated in macrophages and neutrophils, derived from the bone marrow.

VEGFR1 and VEGFR2, together with chemokine CXCL-receptor, expressing angio-genic macrophages, up-regulate VEGF-A in injured peripheral nerves and contribute to angiogenesis and prolonged pain. It has been proposed that VEGF-A gene-related factors may cause peripheral sensitization, leading to neuropathic pain (6). Cancer cells and activated fibroblasts also secrete elevated levels of VEGF- and CXCL-family chemokines, which results in the active recruitment of leukocytes and endothelial cells in tumor vicinity (7).

Another important factor in the pain pathophysiology of pain is specific populations of nerve fibers, which innervate the skeleton. Approximately 80% of the unmyelinated/thinly myelinated sensory nerve fibers, expressing calcitonin gene-related peptide (CGRP) and innervating the periosteum, mineralized bone, and bone marrow, also express tropomyosin receptor kinase A (TrkA). Similarly, the majority of myelinated sensory nerve fibers, which express neurofilament 200 kDa (NF200) and innervate the periosteum, mineralized bone, and bone marrow, also co-express TrkA. In the normal femur, the decreasing order of the relative density of CGRP+, NF200+, and TrkA+ sensory nerve fibers per unit volume is: periosteum> bone marrow> mineralized bone> cartilage, with the respective relative densities being 100:2:0.1:0; this explains why the blockade of TrkA pathway is effective in bone pain management (8).

Since in the normal femur, pain mediators have a 50-fold higher concentration in the periosteum than in bone marrow, bone marrow disease may be associated with lesser pain, compared to mild periosteal involvement. Our patient had multiple bone marrow metastases, seen in the ^18^F-FDG scan, probably causing a minimal periosteal reaction. The cortical bone was only slightly affected, as seen in PET images and more importantly in ^153^Sm-EDTMP post-therapy scintigraphy. Since the pain is mainly mediated by receptors located in the cortical bone, cortical and extensive bone marrow disease together caused more severe pain than a minor cortical skeletal disease.

It has been reported that red marrow absorbed dose after ^153^Sm-EDTMP administration ranges from 2 to 5 Gy (9). We estimated that the absorbed doses in the bone marrow and periosteum were 5.3 Gy and 24.2 Gy in our case, respectively.

In the ^18^F-FDG scan, it is clearly seen that almost all of the metastases were located in the bone marrow (Figure 2). The probable mechanism of pain relief in our case is the ability of β-particles to disrupt the “vicious circle” (8), irradiating the specific populations of nerve fibers that innervate the skeleton, as well as the irradiation of cancer cells, which may lead to the termination of VEGF production and possible production of other growth factors.

The present case explicitly demonstrates that guidelines should not always be literally followed; as a matter of fact, indications should be re-evaluated case by case. Our patient would have been deprived of an effective palliative treatment if the standard approach had been applied. It may be postulated that in our case, the pain relief was due to docetaxel. Pain relief started rather rapidly, which seldom happens with such a low dose of docetaxel. In the follow-up after combined low-dose chemotherapy and radionuclide therapy for skeletal pain, bone marrow toxicity should be carefully evaluated.

Even though this is only a case report with its own limitations, we would like to propose the extensive bone marrow disease accompanied by skeletal pain, without any significant findings in bone scintigraphy, as an additional indication for radionuclide therapy for bone pain palliation. This should be investigated in a larger population.

## References

[1] Brodowicz T, O’Byrne K, Manegold C (2012). Bone matters in lung cancer. Ann Oncol.

[2] Bodei L, Lam M, Chiesa C, Flux G, Brans B, Chiti A (2008). EANM procedure guideline for treatment of refractory metastatic bone pain. Eur J Nucl Med Mol Imaging.

[3] Ziai D, Wagner T, El Badaoui A, Hitzel A, Woillard JB, Melloni B (2013). Therapy response evaluation with FDG-PET/CT in small cell lung cancer:a prognostic and comparison study of the PERCIST and EORTC criteria. Cancer Imaging.

[4] Xanthopoulos EP, Corradetti MN, Mitra N, Fernandes AT, Kim M, Grover S (2013). Impact of PET staging in limited-stage small-cell lung cancer. J Thorac Oncol.

[5] Lipton A (2004). Pathophysiology of bone metastases:how this knowledge may lead to therapeutic intervention. J Support Oncol.

[6] Kiguchi N, Kobayashi Y, Kadowaki Y, Fukazawa Y, Saika F, Kishioka S (2014). Vascular endothelial growth factor signaling in injured nerves underlies peripheral sensitization in neuropathic pain. J Neurochem.

[7] Roodman GD (2004). Mechanisms of bone metastasis. N Engl J Med.

[8] Castañeda-Corral G, Jimenez-Andrade JM, Bloom AP, Taylor RN, Mantyh WG, Kaczmarska MJ (2011). The majority of myelinated and unmyelinated sensory nerve fibers that innervate bone express the tropomyosin receptor kinase A. Neuroscience.

[9] Chappard D, Bouvard B, Baslé MF, Legrand E, Audran M (2011). Bone metastasis:histological changes and pathophysiological mechanisms in osteolytic or osteosclerotic localizations. A review. Morphologie.

[10] Pacilio M, Ventroni G, Basile C, Ialongo P, Becci D, Mango L (2014). Improving the dose-myelotoxicity correlation in radiometabolic therapy of bone metastases with^153^Sm-EDTMP. Eur J Nucl Med Mol Imaging.

